# Synthesis, Biological Evaluation and Molecular Modelling of 2′-Hydroxychalcones as Acetylcholinesterase Inhibitors

**DOI:** 10.3390/molecules21070955

**Published:** 2016-07-22

**Authors:** Sri Devi Sukumaran, Chin Fei Chee, Geetha Viswanathan, Michael J. C. Buckle, Rozana Othman, Noorsaadah Abd. Rahman, Lip Yong Chung

**Affiliations:** 1 Department of Pharmacy, Faculty of Medicine, University of Malaya, 50603 Kuala Lumpur, Malaysia; shivanesri@yahoo.com (S.D.S.); cfchee@yahoo.com (C.F.C.); geethaviswa@gmail.com (G.V.); rozanaothman@um.edu.my (R.O.); 2 Department of Chemistry, Faculty of Science, University of Malaya, 50603 Kuala Lumpur, Malaysia; noorsaadah@um.edu.my

**Keywords:** Alzheimer’s disease, acetylcholinesterase, chalcones, molecular modelling

## Abstract

A series of 2′-hydroxy- and 2′-hydroxy-4′,6′-dimethoxychalcones was synthesised and evaluated as inhibitors of human acetylcholinesterase (AChE). The majority of the compounds were found to show some activity, with the most active compounds having IC_50_ values of 40–85 µM. Higher activities were generally observed for compounds with methoxy substituents in the A ring and halogen substituents in the B ring. Kinetic studies on the most active compounds showed that they act as mixed-type inhibitors, in agreement with the results of molecular modelling studies, which suggested that they interact with residues in the peripheral anionic site and the gorge region of AChE.

## 1. Introduction

Alzheimer’s disease (AD) is a degenerative brain condition characterised by severe memory loss and cognitive impairment. It currently affects more than 30 million people worldwide, and these numbers are set to quadruple by the year 2050 [1]. Following the cholinergic hypothesis, the current first-line treatment for AD is the administration of acetylcholinesterase (AChE) inhibitors, which block the enzymatic hydrolysis of acetylcholine (ACh), resulting in increased levels of the neurotransmitter in the synapses between cholinergic neurons, thereby improving nerve transmission [2]. The agents that have been approved for clinical use to date have limited efficacy and may produce adverse effects, such as nausea, vomiting, diarrhea, dizziness, and weight loss [3]. There is therefore a need for the development of new AChE inhibitors.

Crystallographic studies of AChE complexed with various inhibitors have shown that the enzyme possesses two separate ligand binding sites—a catalytic site (CAS) and a peripheral anionic site (PAS)—which are connected by a narrow gorge [4]. The two key binding residues in the CAS and PAS are aromatic residues, Trp84 and Trp286. The classical hydrolytic role of AChE is performed at the CAS, whereas the PAS has been associated with a number of non-classical roles, including the induction of amyloid β (Aβ) aggregation to form fibrils, which is a key step in the formation of Aβ plaques. Since these plaques are one of the main pathological hallmarks of AD, the use of inhibitors that bind to the PAS and so inhibit both Aβ aggregation and ACh hydrolysis by blocking the approach to the catalytic site has been proposed as a potential disease-modifying therapy [5]. Electron-rich aromatic rings are a common feature of PAS-binding AChE inhibitors, which are believed to stack against the Trp286 residue of the PAS site, as exemplified in the recently-determined crystal structure of human AChE in complex with donepezil [6]. However, in the case of compounds which contain aromatic rings that interact with residues in the CAS or the gorge, electron-withdrawing substituents have been found to increase AChE inhibition in comparison to unsubstituted compounds, especially when they are in the para position [7,8].

Chalcones, or 1,3-diaryl-2-propen-1-ones, are a class of polyphenolic compounds belonging to the flavonoid family which possess a wide range of pharmacological activities, including anti-cancer, anti-inflammatory, and anti-oxidant activities [9]. However, there have been relatively few previous reports of chalcones and their derivatives with AChE inhibitory activity [10,11,12,13,14,15,16], and the effects of different substituents on the phenyl rings have not yet been extensively investigated. In the present work, we chose to study 2′-hydroxychalcones, as compounds with this scaffold have been previously reported to show good activity against AChE with strong selectivity compared to butyrylcholinesterase (BChE) [10]. Furthermore, it has been recently reported that good AChE inhibitory activity has been exhibited by some derivatives of the naturally-occurring 2′-hydroxy-4′,6′-dimethoxychalcone, flavokawain B [15], and that compounds with this substitution pattern in the A ring are non-cytotoxic at 100 µM concentration towards human embryonic kidney (HEK-293) cells [17], which resemble developing neuronal cells [18]. Hence, we decided to synthesise and compare the AChE inhibitory activities of a series of 2′-hydroxy- and 2′-hydroxy-4′,6′-dimethoxychalcones with a range of substituents attached to the B ring. Furthermore, kinetic and molecular modelling studies were carried out on the most active compounds to investigate their mode of inhibition and binding interactions with AChE.

## 2. Results and Discussion

**Scheme 1:**
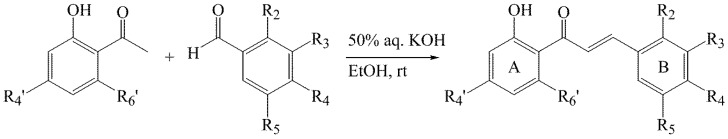
Synthesis of 2′-hydroxychalcones.

2′-hydroxychalcones **1**–**14** were prepared from the corresponding 2′-hydroxyacetophenones and benzaldehydes via the Claisen–Schmidt reaction (Scheme 1). The physicochemical and spectroscopic data obtained from the new compounds **8** and **14** were in agreement with their structures, and those from the known compounds were in agreement with published data. Examination of the predicted Lipinski rule of five parameters of the compounds (Table 1) shows that they all obey both the rule of five and the more restrictive blood-brain permeability considerations (molecular weight < 450, number of hydrogen bond donors (HBD) < 3, log *P* 2–5, polar surface area (PSA) < 90 Å^2^) [19], suggesting that they should be orally active and able to reach the central nervous system.

The inhibitory activity of the prepared 2′-hydroxychalcones at human recombinant AChE was evaluated at 10 µM using Ellman’s colorimetric assay method [20], and the IC_50_ values of the compounds that showed more than 20% inhibition were determined. The results are summarised in Table 2. The majority of the compounds showed some AChE inhibitory activity, with the most active compounds having IC_50_ values in the range of 40–85 µM. This level of activity is comparable to that observed for the standard PAS-binding AChE inhibitor propidium, but somewhat lower than that obtained for the standard CAS-binding inhibitor tacrine. Compounds **9**–**14**, which bear methoxy groups at positions C-4´ and C-6´ in the A ring, were generally more active than the corresponding compounds **1**–**8**, which lack these substituents. This observation is consistent with previous work which has shown the beneficial effect of electron-donating moieties in PAS-binding AChE inhibitors with different scaffolds [21,22]. In contrast, when examining the effect of substitution in the B ring, compounds **7**, **10**, **13**, and **14** with halogen substituents (Br/Cl) showed the greatest activity, although not all the compounds with halogen substituents in the B ring had measurable activity at 10 µM. Such an effect due to halogen substituents has also been previously observed in different series of AChE inhibitors and has been explained in terms of additional hydrogen bonding or dipole-dipole interactions, or increased van der Waals interactions [7,8].

**Figure 1:**
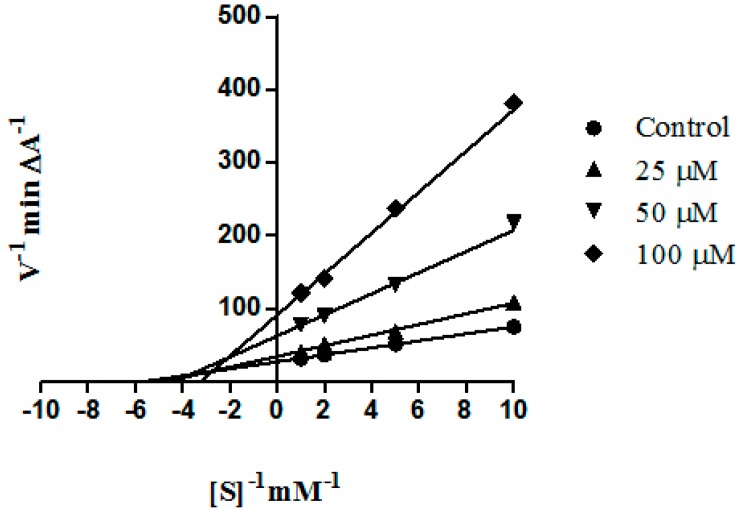
Lineweaver-Burk plot for the inhibition of AChE hydrolysis of acetylthiocholine at different concentrations of compound **10**. Each point is the mean of triplicate determinations.

To investigate the mode of inhibition of the most active compounds and to determine their apparent inhibition constant (*K*_i_ and *K*_i_′) values, kinetic studies were carried out on their steady state inhibition of AChE. For each of the compounds, Lineweaver–Burk plots showed increasing slopes and increasing intercepts at higher inhibitor concentrations and intersections in the second quadrant, above the x-axis and to the left of the y-axis, suggesting that they are all mixed-type inhibitors (see Figure 1). The apparent *K*_i_ and *K*_i_′ values obtained from nonlinear regression analysis are shown in Table 3.

The IC_50_ values of the four compounds that gave the greatest inhibition of AChE were also determined for equine BChE, which is readily available and is considered a good model for human BChE [23], as the equine form has 90% sequence identity compared to the human form. The compounds all showed appreciable BChE inhibitory activity, but generally at lower levels than for AChE, giving selectivities for AChE ranging from 1.1–2.0 (see Table 3). The exception was compound **13**, which showed some selectivity for BChE. Interestingly, BChE has been proposed as a therapeutic target for advanced AD, when BChE takes over the role of ACh hydrolysis from AChE, as neuronal AChE is depleted [24]. Hence, compounds that act as dual inhibitors of both AChE and BChE may also be clinically useful.

**Figure 2:**
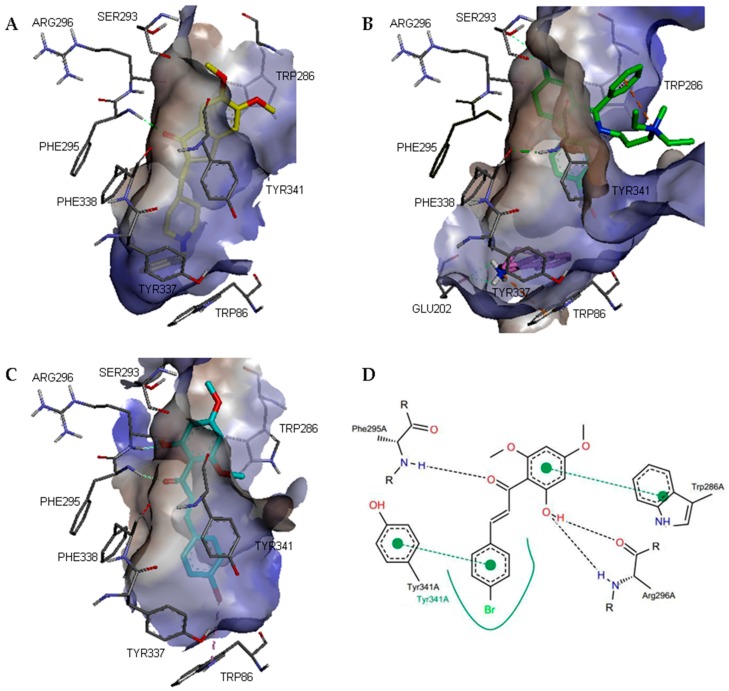
(**A**–**C**) 3D representations of the binding poses of standard and test compounds in complex with human AChE (PDB ID: 4EY7). The hydrophobic surfaces of the interacting residues are shown in blue relief. Ligand–protein interactions are depicted with dotted lines: hydrogen bonds (green), π-cation interactions (orange), π-halogen interactions (purple). (**A**) donepezil; (**B**) propidium and tacrine; (**C**) compound **10**; (**D**) Schematic representation of the binding interactions of compound **10**. Hydrogen bonds and π–π stacking interactions are depicted with black and green dotted lines, respectively. The green curve represents other non-polar interactions.

To investigate possible binding interactions, molecular modelling studies were performed by randomly docking the most active compounds **7**, **10**, **13**, and **14** into human AChE using AutoDock v4.0. The recently-solved X-ray crystal structure of the mixed-type inhibitor, donepezil, with human AChE (PDB ID: 4EY7) [6] was used as the starting model. All the compounds were found to exhibit binding poses similar to those of donepezil occupying the PAS and, to varying extents, the gorge connecting it to the CAS (see Figure 2 and Supplementary Figures S1–S3). Specifically, in the case of compound **10**, aromatic rings A and B formed π–π stacking interactions with Trp286 (in the PAS) and Tyr 341 (at the entrance to the gorge leading to that CAS), respectively. The other three compounds showed π–π stacking or non-polar interactions with one or other of these two residues and/or Tyr337 and Phe338 in the gorge. These interactions with residues in the gorge region may account for the *K*_i_ values obtained from the kinetic studies being less than the *K*_i_′ values—i.e., the competitive part of the mixed-type inhibition was more pronounced than the uncompetitive part. Furthermore, in the case of compounds **7** and **10**, the carbonyl oxygen atom and the ortho hydroxyl group on the A ring were also observed to form hydrogen bonds with backbone atoms from acyl pocket residues, Phe295 (2.8–3.0 Å) and Arg296 (2.7–2.9 Å), respectively. This may be one of the reasons to account for the greater activity of these two compounds, as compounds **13** and **14** only interacted with Arg296. Hydrogen bonds to Phe295 have previously been observed in crystal structures of AChE in complex with donepezil and other inhibitors [8,25,26]. The modelling studies also suggested that the beneficial effect of the halogen substituents in the active compounds might be due to interactions with aromatic residues in the CAS and gorge. Such interactions have previously been observed in a number of other protein–ligand complexes [27].

Molecular modelling studies were also performed by randomly docking the most active compounds **7**, **10**, **13**, and **14** into a model derived from the recently-solved X-ray crystal structure of tacrine in complex with human BChE (PDB ID: 4BDS) [28]. Although all four compounds were found to bind, the calculated binding energy was 1.1–1.8 kcal/mol lower for AChE compared to BChE. These results are consistent with the slight selectivity for AChE compared to BChE that was observed for three out of the four most active compounds in the inhibition studies.

## 3. Materials and Methods

### 3.1. General Information

All melting points were taken on a Stuart melting point apparatus SMP30 (Staffordshire, UK). NMR spectra were obtained using Jeol ECA 400 (400 MHz) and EX 270 (270 MHz) NMR spectrometers (Jeol Ltd., Akishima, Tokyo, Japan) with tetramethylsilane as the internal standard. All chemical shifts are reported in ppm. MS analysis was performed on an Agilent 6500 series accurate mass Q-TOF (Agilent Technologies, Santa Clara, CA, USA) Analytical thin-layer chromatography (TLC) was carried out on pre-coated aluminum silica gel sheets (Kieselgel 60 F254) from Merck (Darmstadt, Germany). Column chromatography was performed with silica gel 60 (230–400 mesh) from Merck. Human recombinant AChE and equine BChE were purchased from Sigma-Aldrich (St. Louis, MO, USA). All other chemicals were purchased from Sigma-Aldrich or Merck and were of analytical grade. The Lipinski rule of five parameters of the synthesised compounds were determined using Discovery Studio v3.1 (Accelrys Inc., San Diego, CA, USA).

### 3.2. Synthesis of Chalcones ***1**–**14***

A mixture of the corresponding acetophenone (1 equiv) and benzaldehyde (1 equiv) in EtOH (5 mL/1 mmol of acetophenone) was stirred at room temperature for 30 min. Then, a solution of 50% *w*/*w* aqueous KOH (1 mL/1 mmol) was added. The reaction mixture was stirred at rt until all benzaldehyde was consumed when monitored on TLC. Afterwards, the mixture was poured into ice-water acidified with 3 N HCl. In cases in which the chalcones precipitated, they were filtered, purified using column chromatography, and crystallised from ethanol (yields: 65%–90%).

*2′-Hydroxychalcone* (**1**). Yellow crystals, mp 89–90 °C, lit. 89–90 °C [29]; for NMR data see ref. [30].

*2′-Hydroxy-4-methylchalcone* (**2**) Yellow needles, mp 117–118 °C, lit. 116–117 °C [31]; for NMR data see ref. [31].

*4-Bromo-2′-hydroxychalcone* (**3**). Yellow needles, mp 138–139 °C, lit. 138–139 °C [32]; ^1^H-NMR (400 MHz, CDCl_3_): δ 12.67 (s, 1H, OH), 7.80 (dd, *J* = 8.0, 1.5 Hz, 1H, H-6′), 7.73 (d, *J* = 16.0 Hz, 1H, H_β_), 7.53 (d, *J* = 16.0 Hz, 1H, H_α_), 7.47 (d, *J* = 8.1 Hz, 2H, H-2, H-6), 7.41 (d, *J* = 7.8 Hz, 2H, H-3, H-5), 7.40 (m, 1H, H-4′), 6.94 (dd, *J* = 8.0, 1.5 Hz, 1H, H-3′), 6.85 (m, 1H, H-5′); ^13^C-NMR (100 MHz, CDCl_3_): δ 193.44, 163.65, 143.96, 136.56, 133.53, 132.31, 129.97, 129.62, 125.27, 120.71, 119.94, 118.92, 118.70.

*4-Chloro-2′-hydroxychalcone* (**4**). Yellow needles, mp 145–146 °C, lit. 148–151 °C [29]; for NMR data see ref. [31].

*2′-Hydroxy-4-(dimethylamino)chalcone* (**5**). Orange powder, mp 170–171 °C, lit. 172 °C [33]; for NMR data see ref. [33].

*2′-Hydroxy-4-methoxychalcone* (**6**). Orange needles, mp 92–93 °C, lit. 92–94 °C [29]; for NMR data see ref. [31].

*2-Bromo-2′-hydroxychalcone* (**7**). Yellow needles, mp 104–105 °C, lit. 103–104 °C [32]; ^1^H-NMR (400 MHz, CDCl_3_): δ 12.63 (s, 1H, OH), 8.12 (d, *J* = 15.3 Hz, 1H, H_β_), 7.77 (dd, *J* = 8.1, 1.8 Hz, 1H, H-6′), 7.61 (dd, *J* = 8.1, 1.8 Hz, 1H, H-3), 7.51 (dd, *J* = 8.2, 1.9 Hz, 1H, H-4′), 7.44 (d, *J* = 15.4 Hz, 1H, H_α_), 7.38 (m, 1H, H-5), 7.25 (t, *J* = 7.3 Hz, 1H, H-6), 7.13 (m, 1H, H-4), 6.90 (dd, *J* = 8.0, 1.8 Hz, 1H, H-3′), 6.82 (m, 1H, H-5′); ^13^C-NMR (100 MHz, CDCl_3_): δ 193.42, 163.66, 143.65, 136.61, 134.70, 133.67, 131.69, 129.78, 128.03, 127.81, 126.16, 122.93, 119.90, 118.97, 118.68.

*3,5-Dichloro-2,2′-dihydroxychalcone* (**8**). Yellow powder, mp 125–126 °C. ^1^H-NMR (400 MHz, DMSO-*d*_6_): δ 12.48 (s, 1H, OH), 10.43 (s, 1H, OH), 8.26 (d, *J* = 7.8 Hz, 1H, H-6′), 8.12 (d, *J* = 15.5 Hz, 1H, H_β_), 8.06 (d, *J* = 15.5 Hz, 1H, H_α_), 7.57 (m, 1H, H-4′), 7.55 (d, *J* = 2.3 Hz, 1H, H-4), 7.53 (d, *J* = 2.3 Hz, 1H, H-6), 7.00 (m, 1H, H-3′), 6.98 (m, 1H, H-5′); ^13^C-NMR (100 MHz, DMSO-*d*_6_): δ 193.99, 162.45, 151.89, 138.05, 136.93, 131.43, 131.38, 126.80, 126.19, 124.59, 123.82, 123.59, 121.11, 119.60, 118.20; HREIMS *m/z* 309.0085 [M + H]^+^ (calculated for C_15_H_11_O_3_Cl_2_, 309.0080).

*2′-Hydroxy-4′,6′-dimethoxy-4-methylchalcone* (**9**). Orange needles, mp 130–131 °C, lit. 132–133 °C [31]; for NMR data see ref. [31].

*4-Bromo-2′-hydroxy-4′,6′-dimethoxychalcone* (**10**). Orange powder, mp 150–151 °C, lit. 150–151 °C [34]; for NMR data see ref. [34].

*2′-Hydroxy-4′,6′-dimethoxy-4-(dimethylamino)chalcone* (**11**). Brown crystals, mp 163–165 °C; for NMR data see ref. [17].

*2′-Hydroxy-4,4′,6′-trimethoxychalcone* (**12**). Orange powder, mp 112–113 °C, lit: 112 °C [35]; for NMR data see ref. ref. [35].

*2-Bromo-2′-hydroxy-4′,6′-dimethoxychalcone* (**13**). Orange powder, mp 147–148 °C, lit. 146–147 °C [36]; for NMR data see ref. [36].

*3,5-Dichloro-2,2′-dihydroxy-4′,6′-dimethoxychalcone* (**14**). Yellow needles, mp 192–193 °C. ^1^H-NMR (400 MHz, DMSO-*d_6_*): δ 13.22 (s, 1H, OH), 7.84 (d, *J* = 15.6 Hz, 1H, H_β_), 7.80 (d, *J* = 15.6 Hz, 1H, H_α_), 7.68 (d, *J* = 2.4 Hz, 1H, H-4), 7.58 (d, *J* = 2.4 Hz, 1H, H-6), 6.15 (s, 1H, H-5′), 6.13 (s, 1H, H-3′), 3.88, 3.83 (s, 3H each, OCH_3_); ^13^C-NMR (100 MHz, CDCl_3_): δ 192.87, 166.07, 165.66, 162.35, 151.55, 136.15, 130.69, 130.46, 127.21, 126.68, 124.42, 123.46, 107.01, 94.36, 91.56, 56.70, 56.16; HREIMS *m/z* 369.0303 [M + H]^+^ (calculated for C_17_H_15_O_5_Cl_2_, 369.0297).

### 3.3. Enzyme Inhibition Studies

AChE and BChE inhibition studies were performed in 96 well plates by the method of Ellman, et al. [20]. 110 μL of sodium phosphate buffer (pH 8.0) was added to each well followed by 20 μL of test compound, 50 μL of 5,5′-dithiobis-(2-nitrobenzoic acid) (DTNB) (0.126 mM) and 20 μL of AChE or BChE (0.15 units/mL). The mixture was preincubated for 20 min at 37 °C before addition of 50 μL (0.120 mM) of the substrate, acetylthiocholine (ATC) iodide or butyrylthiocholine (BTC) iodide (depending on the enzyme). The hydrolysis of ATC or BTC was monitored using an Infinite^®^ M200 PRO multimode reader (Tecan Group Ltd., Männedorf, Switzerland) by measuring the absorbance due to yellow 5-thio-2-nitrobenzoate anion at 412 nm every 30 s for 25 min. Each assay was performed in triplicate. Propidium iodide and tacrine were used as standard inhibitors. The percentage of inhibition was calculated using the expression:

(*E–S*)/*E* × 100

where *E* is the activity of the enzyme without test compound and *S* is the activity of the enzyme with test compound.

IC_50_ values were obtained from concentration-inhibition experiments by nonlinear regression analysis using PRISM^®^ v5.0 (GraphPad Inc., San Diego, CA, USA).

Kinetic characterisation of AChE inhibition was performed under similar conditions to the AChE assay using different concentrations of test compounds (0–100 μM) at substrate concentrations ranging from 0.1 mM to 1.0 mM. Each assay was performed in triplicate. The mode of inhibition was investigated using Lineweaver–Burk plots of 1/initial reaction rate (1/V) against 1/substrate concentration (1/[S]). Apparent inhibition constant (*K*_i_ and *K*_i_′) values were determined using the nonlinear regression mixed-model inhibition method in PRISM^®^ v5.0 (GraphPad Inc.).

### 3.4. Molecular Modelling Studies

The X-ray crystal structures of human AChE and BChE in complex with donepezil and tacrine, respectively, were retrieved from the Protein Data Bank (PDB IDs: 4EY7 and 4BDS). Ligands and water molecules were removed using Discovery Studio v3.1. Hydrogen atoms were added and double coordinates were corrected using Hyperchem Pro v6.0 (Hypercube Inc., Gainesville, FL, USA). Hydrogen atoms were again added, non-polar hydrogen atoms were merged, and missing atoms were repaired using AutoDock Tools v4 [37]. Gasteiger charges were added and AutoDock v4.0 type atoms were assigned to the protein. Three dimensional structural models of the test compounds were built using Chem Bio-3D v13 (CambridgeSoft, Cambridge, MA, USA) and saved in MOL2 format. The compounds were then prepared in protonated form, where appropriate for physiological pH, and minimised using Hyperchem Pro v6.0 (geometry optimisation using MM+) to give the lowest energy conformation. Test compounds were randomly docked into the protein structures with AutoDock v4.0 using a hybrid Lamarckian Genetic Algorithm [38], with an initial population size of 150 and a maximum number of 2,500,000 energy evaluations. The grid box was set to cover the entire protein with a spacing of 0.375 Å. The root mean square deviation (RMSD) tolerance was set to 2.0 Å for the clustering of docked results and the docked pose of each ligand was selected on the basis of free energy of binding and cluster analysis. Ligand-protein interactions were analysed using Discovery Studio v3.1 and PoseView [39].

## 4. Conclusions

In conclusion, a series of 2′-hydroxychalcones was synthesised and evaluated as inhibitors of human AChE. Compounds with methoxy substituents on the A ring and halogen substituents on the B ring were generally found to be more potent, with activities that are comparable to the standard PAS-binding AChE inhibitor, propidium. Kinetic studies on the most active compounds showed that they act as mixed-type inhibitors. This finding was consistent with molecular modelling studies, which suggested that the compounds interact with residues in the PAS and gorge region of AChE. Further investigations into the use of 2′-hydroxychalcone derivatives as potential disease-modifying drug candidates for the treatment of AD are in progress.

## Figures and Tables

**Table 1:** Lipinski rule of five parameters for 2′-hydroxychalcones **1**–**14**.

Compound	MW	HBD	HBA	*A* log *P*	PSA/Å^2^
**1**	224.26	1	2	3.46	38.12
**2**	238.28	1	2	3.95	38.12
**3**	303.15	1	2	4.21	38.12
**4**	258.70	1	2	4.12	38.12
**5**	267.32	1	3	3.62	41.49
**6**	254.28	1	3	3.44	47.05
**7**	303.15	1	2	4.21	38.12
**8**	309.14	2	3	4.55	58.93
**9**	298.33	1	4	3.91	55.98
**10**	363.20	1	4	4.18	55.98
**11**	327.37	1	5	3.59	59.33
**12**	314.33	1	5	3.41	64.91
**13**	363.20	1	4	4.18	55.98
**14**	369.20	2	5	4.51	76.79
Donepezil	379.49	0	4	4.57	38.51

MW: molecular weight; *A* log *P*: logarithm of octanol–water partition coefficient; HBD: number of hydrogen bond donors; HBA: number of hydrogen bond acceptors; PSA: polar surface area.

**Table 2:** Substituent patterns and human acetylcholinesterase (AChE) inhibitory activity of 2′-hydroxychalcones **1**–**14**.

Compound	R_4´_	R_6´_	R_2_	R_3_	R_4_	R_5_	% Inhibition at 10 µM ^a^	IC_50_/µM ^b^
**1**	H	H	H	H	H	H	11	nd ^c^
**2**	H	H	H	H	Me	H	na ^d^	nd ^c^
**3**	H	H	H	H	Br	H	na ^d^	nd ^c^
**4**	H	H	H	H	Cl	H	na ^d^	nd ^c^
**5**	H	H	H	H	NMe_2_	H	28	266 ± 48
**6**	H	H	H	H	OMe	H	6	nd ^c^
**7**	H	H	Br	H	H	H	38	55 ± 12
**8**	H	H	OH	Cl	H	Cl	na ^d^	nd ^c^
**9**	OMe	OMe	H	H	Me	H	23	354 ± 33
**10**	OMe	OMe	H	H	Br	H	30	41 ± 13
**11**	OMe	OMe	H	H	NMe_2_	H	5	nd ^c^
**12**	OMe	OMe	H	H	OMe	H	7	nd ^c^
**13**	OMe	OMe	Br	H	H	H	23	85 ± 16
**14**	OMe	OMe	OH	Cl	H	Cl	51	73 ± 22
Propidium ^e^							50	11 ± 3
Tacrine ^f^							91	0.19 ± 0.04

^a^ Values are expressed as mean of triplicate; ^b^ Values are expressed as mean ± SEM; ^c^ Not determined; ^d^ No activity observed at 10 µM; ^e^ Standard PAS-binding AChE inhibitor; ^f^ Standard CAS-binding AChE inhibitor.

**Table 3:** Apparent human AChE inhibition constant (*K*_i_ and *K*_i_′) values, equine butyrylcholinesterase (BChE) inhibitory activity and selectivity for AChE for the most active compounds.

Compound	Human AChE		Equine BChE	Selectivity
	*K*_i_ /µM	*K*_i_′ /µM	IC_50_ /µM ^a^	for AchE ^b^
**7**	40	59	108 ± 18	2.0
**10**	31	47	59 ± 12	1.4
**13**	113	165	71 ± 14	0.8
**14**	56	92	81 ± 18	1.1

^a^ Values are expressed as mean ± SEM; ^b^ Selectivity is defined as IC_50_ (BChE)/IC_50_ (AChE); values for IC_50_ (AChE) are shown in Table 2.

## Supplementary Materials

Supplementary materials can be accessed at: http://www.mdpi.com/1420-3049/21/7/955/s1.
