# Supplementary material for: Synthesis, Biological Evaluation and Molecular Modelling of 2′-Hydroxychalcones as Acetylcholinesterase Inhibitors
Source: Molecules. 2016 Jul 22;21(7):955. doi: 10.3390/molecules21070955 (PMC6273369; doi:10.3390/molecules21070955)
Supplement: Supplementary file 1 [file molecules-21-00955-s001.pdf]

Supplementary Materials: Synthesis, Biological Evaluation and Molecular Modelling of 2′-Hydroxychalcones as Acetylcholinesterase Inhibitors

Sri Devi Sukumaran, Chin Fei Chee, Geetha Viswanathan, Michael J. C. Buckle, Rozana Othman, Noorsaadah Abd. Rahman and Lip Yong Chung

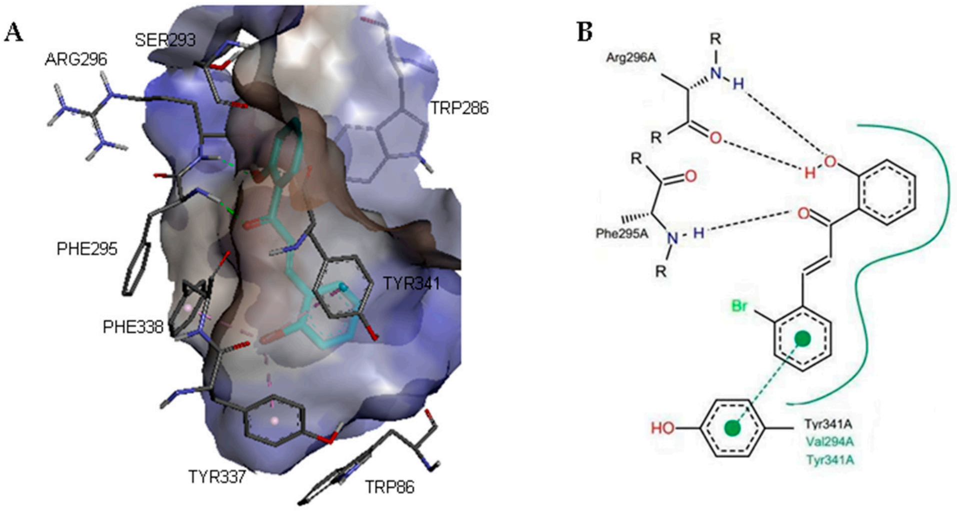

Figure S1. Representations of compound **7** in complex with human AChE (PDB ID: 4EY7). (**A**) 3D representation of the binding pose. The hydrophobic surfaces of the interacting residues are shown in blue relief. Hydrogen bonds and π-halogen interactions are depicted with green and purple dotted lines, respectively; (**B**) Schematic representation of the binding interactions. Hydrogen bonds and π-π stacking interactions are depicted with black and green dotted lines, respectively. The green curve represents other non-polar interactions.

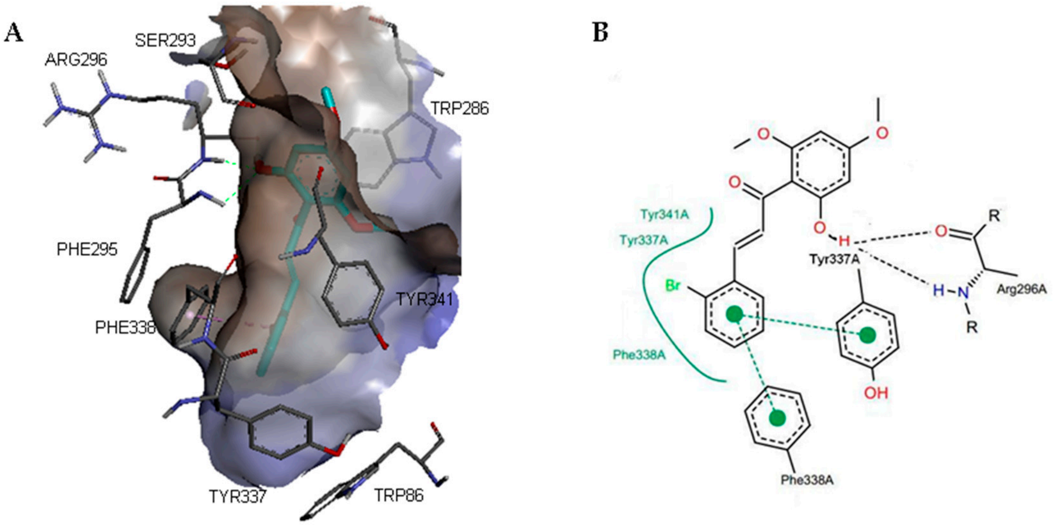

Figure S2. Representations of compound **13** in complex with human AChE (PDB ID: 4EY7). (**A**) 3D representation of the binding pose. The hydrophobic surfaces of the interacting residues are shown in blue relief. Hydrogen bonds and π-halogen interactions are depicted with green and purple dotted lines, respectively; (**B**) Schematic representation of the binding interactions. Hydrogen bonds and π-π stacking interactions are depicted with black and green dotted lines, respectively. The green curve represents other non-polar interactions.

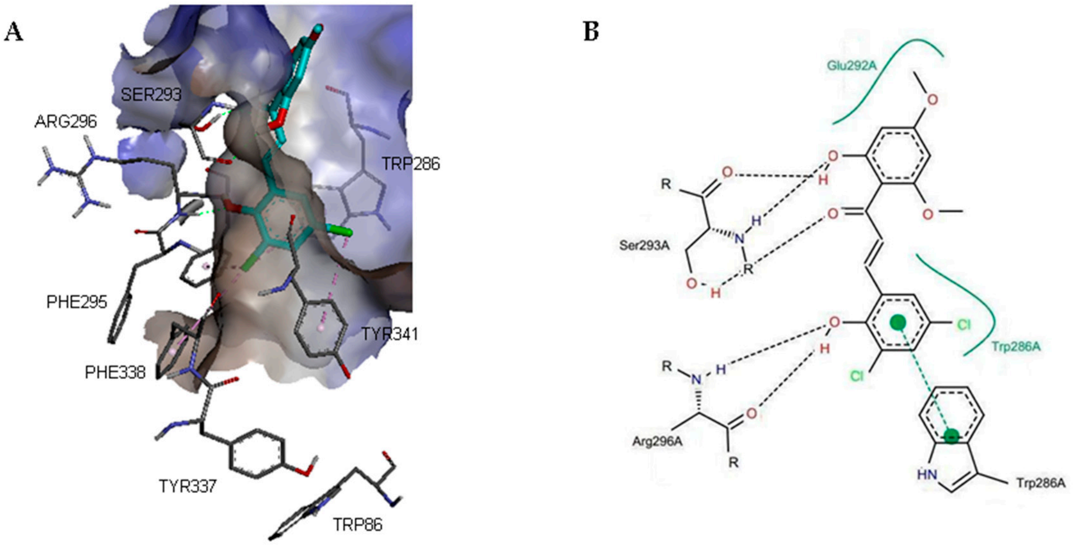

Figure S3. Representations of compound **14** in complex with human AChE (PDB ID: 4EY7). (**A**) 3D representation of the binding pose. The hydrophobic surfaces of the interacting residues are shown in blue relief. Hydrogen bonds and π-halogen interactions are depicted with green and purple dotted lines, respectively; (**B**) Schematic representation of the binding interactions. Hydrogen bonds and π-π stacking interactions are depicted with black and green dotted lines, respectively. The green curve represents other non-polar interactions.

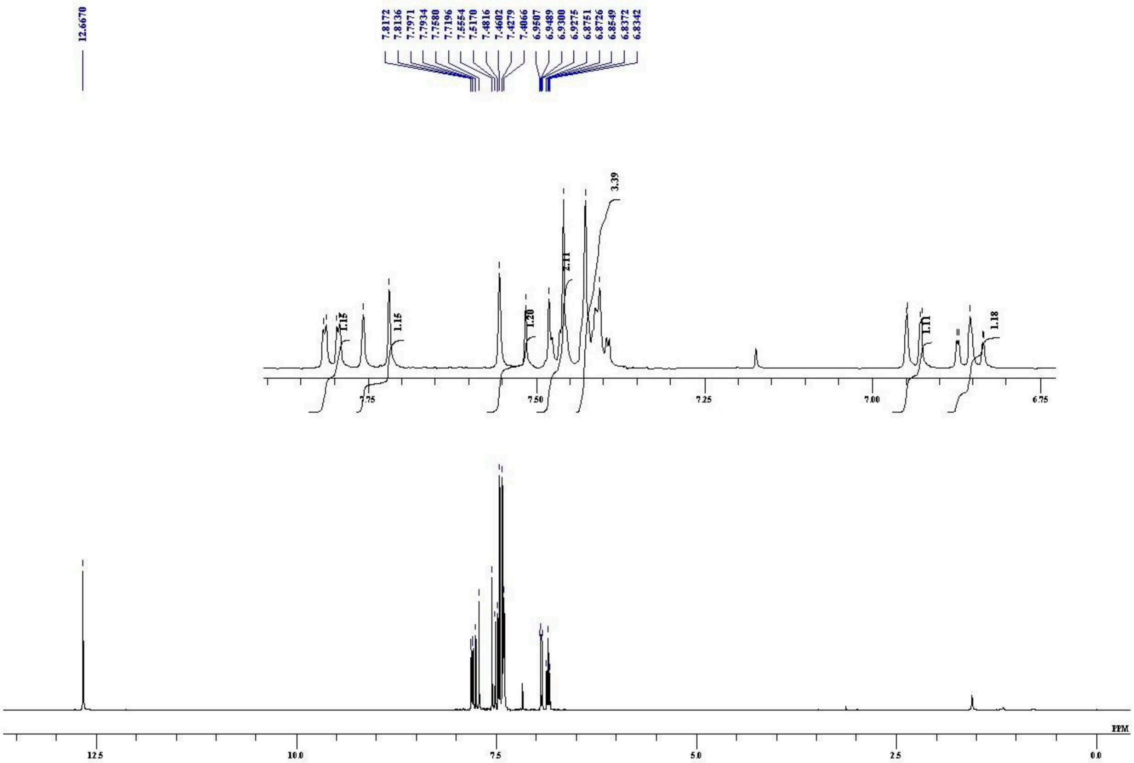

Figure S4. *Cont.*

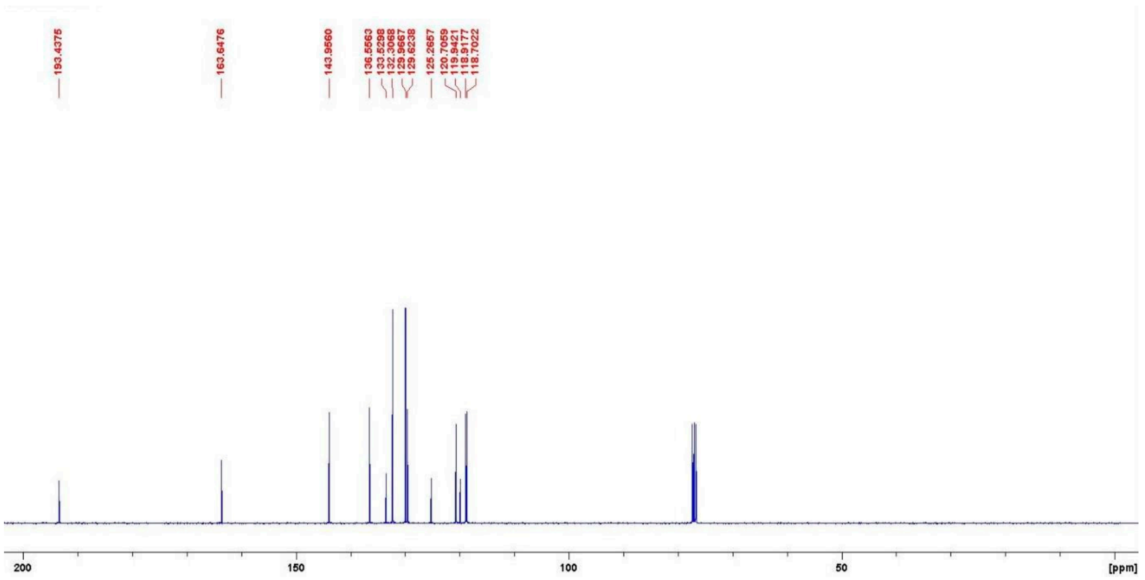

Figure S4. ^{1}H-NMR and ^{13}C-NMR spectra of 4-Bromo-2′-hydroxychalcone (**3**).

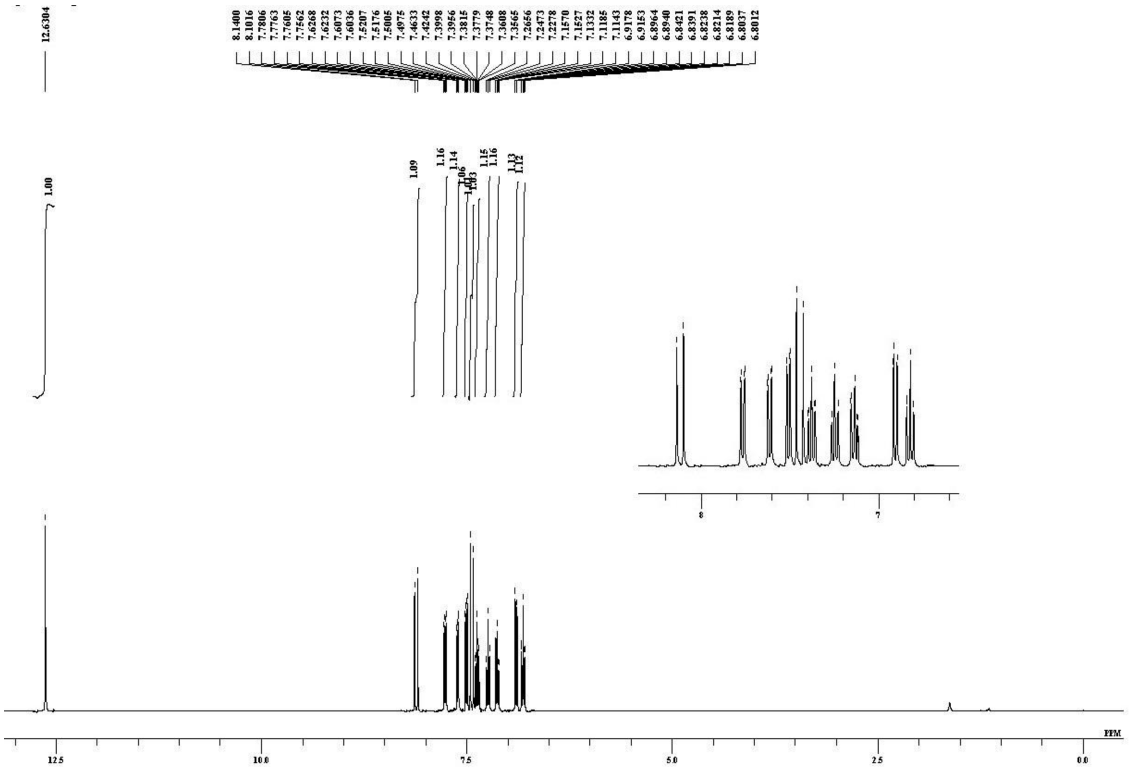

Figure S5. *Cont.*

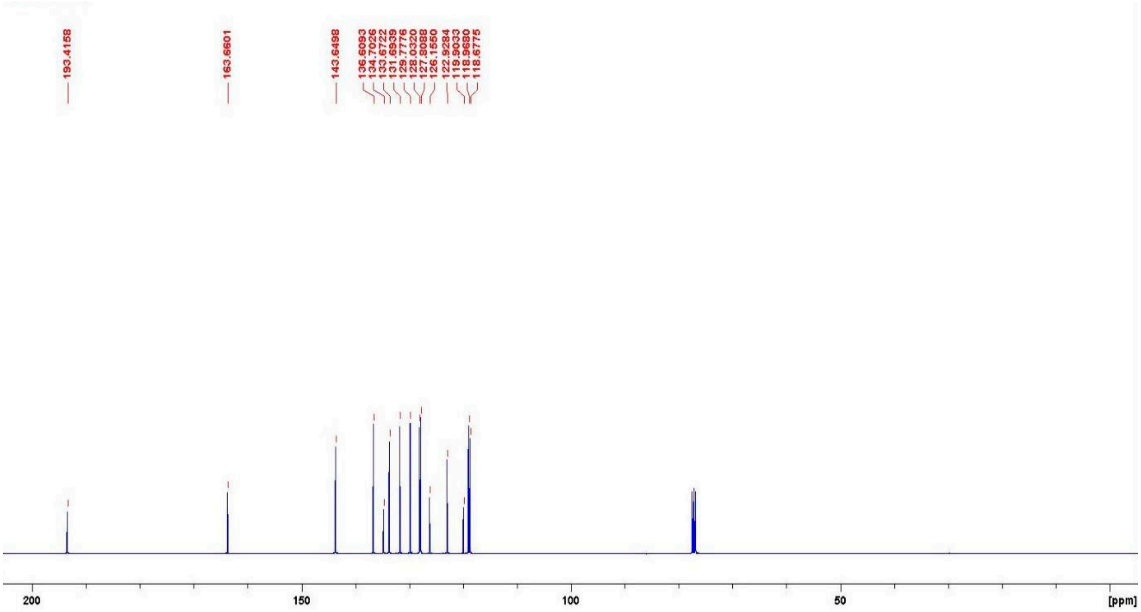

Figure S5. ^{1}H-NMR and ^{13}C-NMR spectra of 2-Bromo-2′-hydroxychalcone (**7**).

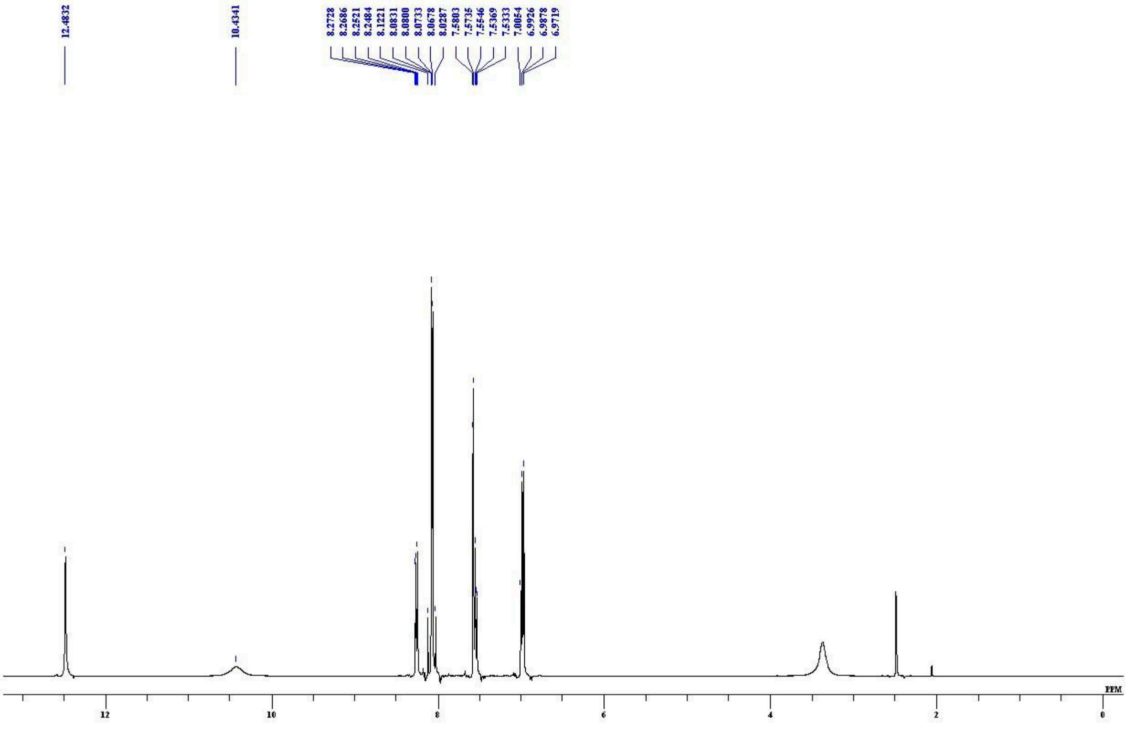

Figure S6. *Cont.*

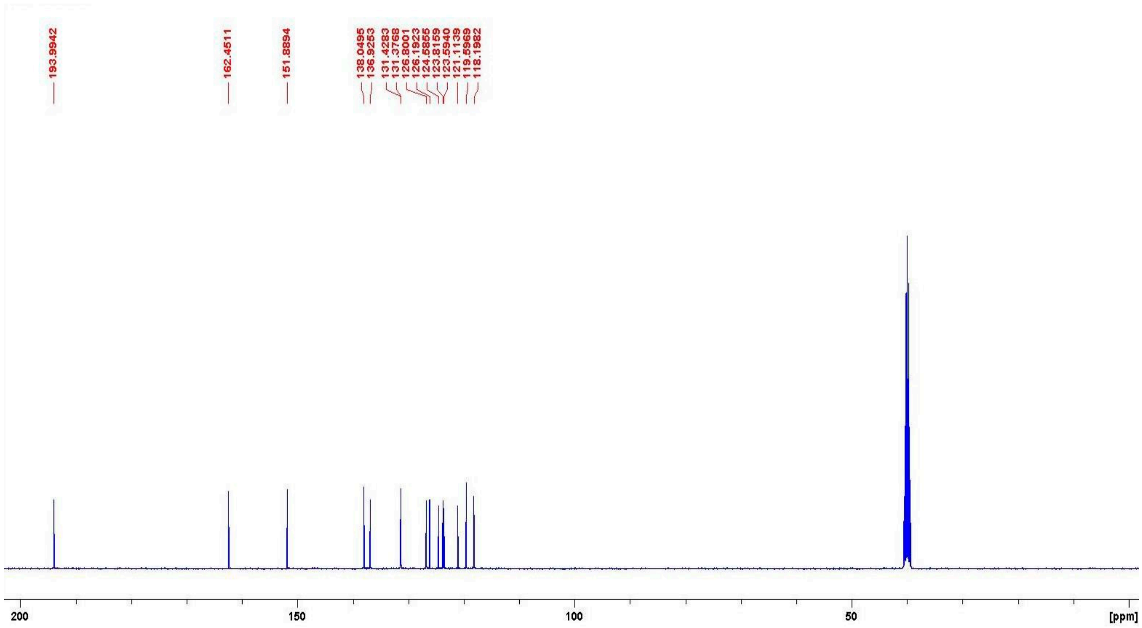

Figure S6. ^{1}H-NMR and ^{13}C-NMR spectra of 3,5-Dichloro-2,2′-dihydroxychalcone (**8**).

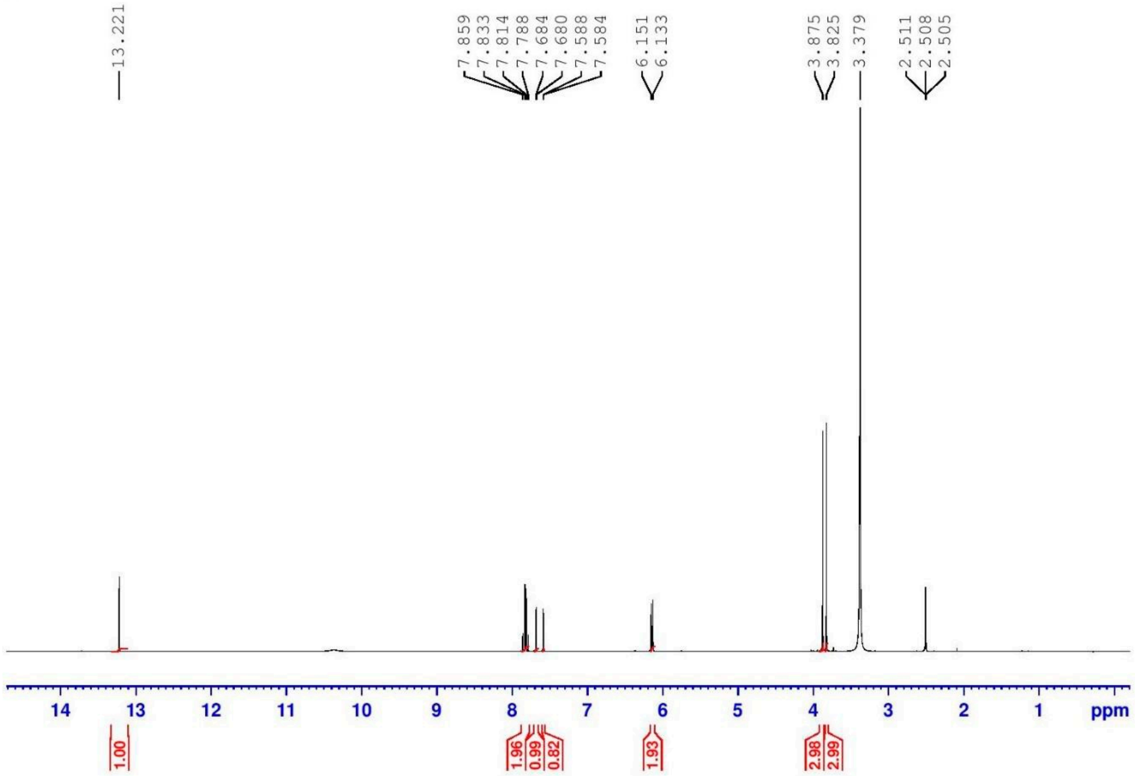

Figure S7. *Cont.*

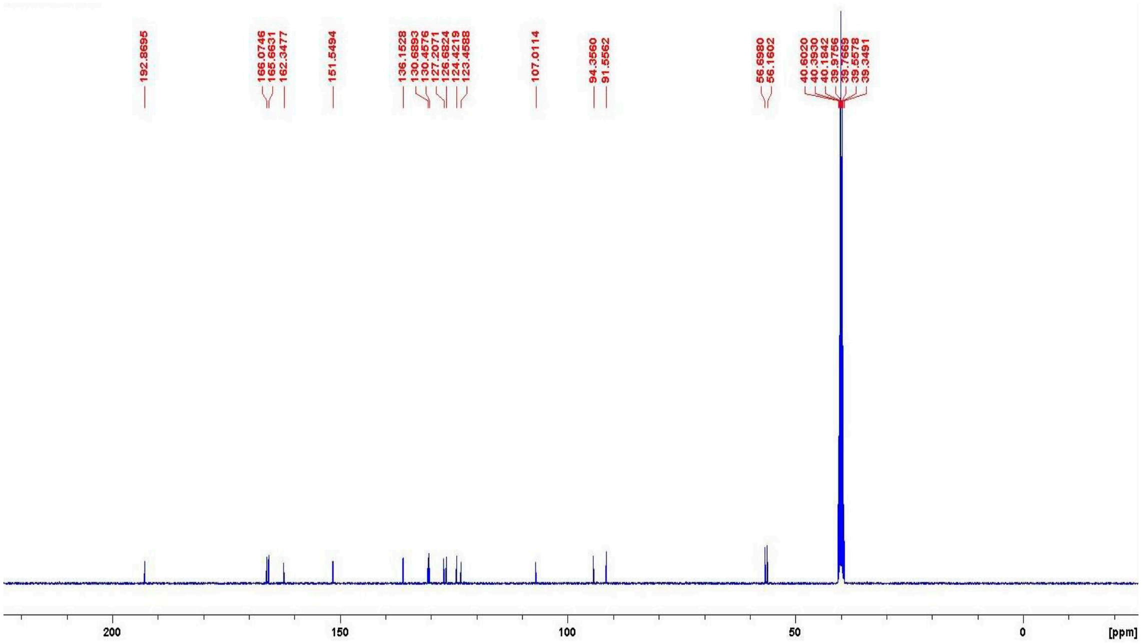

Figure S7. ^{1}H-NMR and ^{13}C-NMR spectra of 3,5-Dichloro-2,2′-dihydroxy-4′,6′-dimethoxychalcone (**14**).
